# Supplementary material for: Cognitive costs and gait parameters during single- and dual-task conditions: A comparative study in individuals with and without non-specific neck pain
Source: PLoS One. 2025 May 20;20(5):e0318963. doi: 10.1371/journal.pone.0318963 (PMC12091795; doi:10.1371/journal.pone.0318963)
Supplement: Attachment — Submitted filename: PONE-D-25-03088.pdf [file pone.0318963.s002.pdf]

PLOS ONE

Cognitive Costs and Gait Parameters During Single- and Dual-Task Conditions: A Comparative Study in Individuals With and Without Non-Specific Neck Pain

--Manuscript Draft--

**Manuscript Number:**	PONE-D-25-03088
**Article Type:**	Research Article
**Full Title:**	Cognitive Costs and Gait Parameters During Single- and Dual-Task Conditions: A Comparative Study in Individuals With and Without Non-Specific Neck Pain
**Short Title:**	Cognitive Costs and Gait in Neck Pain
**Corresponding Author:**	Ibrahim M. Moustafa<br>University of Sharjah<br>Sharjah, Sharjah UNITED ARAB EMIRATES
**Keywords:**	Non-specific neck pain (NSNP); dual-task performance; Cognitive load; gait parameters; pain intensity; cognitive cost
**Abstract:**	Background<br><br>The high incidence of non-specific neck pain (NSNP) among students, commonly associated with prolonged sedentary behavior and extensive use of electronic devices, highlights the need to assess its impact on cognitive and motor functions. This study aims to evaluate the effects of NSNP on dual-task performance by analyzing gait parameters and cognitive performance during both single-task and dual-task conditions.<br><br>Methods<br><br>Forty-five participants with NSNP and forty-five age-matched controls were assessed using an optical motion-capture system. Participants underwent gait assessments during both single-task (without cognitive load) and dual-task conditions, in which the cognitive tasks involved simple mathematical computations.<br><br>Results<br><br>Results revealed that, under single-task conditions, differences in gait parameters between groups were not statistically significant. However, under dual-task conditions, participants with NSNP exhibited significant impairments in gait parameters and higher cognitive costs ($p < 0.05$). Correlation analysis indicated that pain intensity was significantly associated with cognitive cost and gait parameter alterations during dual-task conditions ($p < 0.05$).<br><br>Conclusions<br><br>These findings suggest that NSNP significantly elevates the cognitive effort required during dual tasking. These finding emphasizes on the need for interventions to alleviate neck pain and improve both physical and cognitive health.
**Order of Authors:**	Ibrahim M. Moustafa<br>Shorouk Abu-Ghosh<br>Amal Ahbouch<br>Shima Abdollah Mohammad Zadeh<br>Iman Khowailed<br>Meeyoung Kim
**Additional Information:**	
**Question**	**Response**

Powered by Editorial Manager® and ProduXion Manager® from Aries Systems Corporation

**Financial Disclosure**<br><br>Enter a financial disclosure statement that describes the sources of funding for the work included in this submission. Review the submission guidelines for detailed requirements. View published research articles from *PLOS ONE* for specific examples.<br><br>This statement is required for submission and **will appear in the published article** if the submission is accepted. Please make sure it is accurate.<br><br>**Funded studies**<br>Enter a statement with the following details:<br>• Initials of the authors who received each award<br>• Grant numbers awarded to each author<br>• The full name of each funder<br>• URL of each funder website<br>• Did the sponsors or funders play any role in the study design, data collection and analysis, decision to publish, or preparation of the manuscript?<br><br>Did you receive funding for this work?	The author(s) received no specific funding for this work.
**Competing Interests**<br><br>Use the instructions below to enter a competing interest statement for this submission. On behalf of all authors, disclose any competing interests that could be perceived to bias this work—acknowledging all financial support and any other relevant financial or non-financial competing interests.<br><br>This statement is required for submission and **will appear in the published article** if the submission is accepted. Please make sure it is accurate and that any funding sources listed in your Funding Information later in the submission form are also declared in your Financial Disclosure statement.	The authors have declared that no competing interests exist.

View published research articles from *PLOS ONE* for specific examples.<br><br>**NO authors have competing interests**<br><br>Enter: *The authors have declared that no competing interests exist.*<br><br>**Authors with competing interests**<br><br>Enter competing interest details beginning with this statement:<br><br>*I have read the journal's policy and the authors of this manuscript have the following competing interests: [insert competing interests here]*<br><br>* typeset	
**Ethics Statement**<br><br>Enter an ethics statement for this submission. This statement is required if the study involved:<br><br>• Human participants<br>• Human specimens or tissue<br>• Vertebrate animals or cephalopods<br>• Vertebrate embryos or tissues<br>• Field research<br><br>Write "N/A" if the submission does not require an ethics statement.<br><br>General guidance is provided below. Consult the submission guidelines for detailed instructions. **Make sure that all information entered here is included in the Methods section of the manuscript.**	The study received ethical approval from the University of Sharjah's College of Health Sciences (Ethical approval number: REC-23-05-11-03-S)

Powered by Editorial Manager® and ProduXion Manager® from Aries Systems Corporation

**Format for specific study types**<br><br>**Human Subject Research (involving human participants and/or tissue)**<br>• Give the name of the institutional review board or ethics committee that approved the study<br>• Include the approval number and/or a statement indicating approval of this research<br>• Indicate the form of consent obtained (written/oral) or the reason that consent was not obtained (e.g. the data were analyzed anonymously)<br><br>**Animal Research (involving vertebrate animals, embryos or tissues)**<br>• Provide the name of the Institutional Animal Care and Use Committee (IACUC) or other relevant ethics board that reviewed the study protocol, and indicate whether they approved this research or granted a formal waiver of ethical approval<br>• Include an approval number if one was obtained<br>• If the study involved *non-human primates*, add *additional details* about animal welfare and steps taken to ameliorate suffering<br>• If anesthesia, euthanasia, or any kind of animal sacrifice is part of the study, include briefly which substances and/or methods were applied<br><br>**Field Research**<br><br>Include the following details if this study involves the collection of plant, animal, or other materials from a natural setting:<br>• Field permit number<br>• Name of the institution or relevant body that granted permission	
**Data Availability**<br><br>Authors are required to make all data underlying the findings described fully available, without restriction, and from the time of publication. PLOS allows rare exceptions to address legal and ethical concerns. See the PLOS Data Policy and FAQ for detailed information.	Yes - all data are fully available without restriction

A Data Availability Statement describing where the data can be found is required at submission. Your answers to this question constitute the Data Availability Statement and **will be published in the article**, if accepted.<br><br>**Important:** Stating 'data available on request from the author' is not sufficient. If your data are only available upon request, select 'No' for the first question and explain your exceptional situation in the text box.<br><br>Do the authors confirm that all data underlying the findings described in their manuscript are fully available without restriction?	
**Describe where the data may be found in full sentences. If you are copying our sample text, replace any instances of XXX with the appropriate details.**<br><br>• If the data are **held or will be held in a public repository**, include URLs, accession numbers or DOIs. If this information will only be available after acceptance, indicate this by ticking the box below. For example: *All XXX files are available from the XXX database (accession number(s) XXX, XXX.*).<br>• If the data are all contained **within the manuscript and/or Supporting Information files**, enter the following: *All relevant data are within the manuscript and its Supporting Information files.*<br>• If neither of these applies but you are able to provide **details of access elsewhere**, with or without limitations, please do so. For example:<br><br>*Data cannot be shared publicly because of [XXX]. Data are available from the XXX Institutional Data Access / Ethics Committee (contact via XXX) for researchers who meet the criteria for access to confidential data.*<br><br>*The data underlying the results presented in the study are available from (include the name of the third party*	The data underlying the results presented in this study are available from the corresponding author, Prof. Ibrahim M. Moustafa (iabuamr@sharjah.ac.ae), upon request.

and contact information or URL).<br>• This text is appropriate if the data are owned by a third party and authors do not have permission to share the data.<br><br>* typeset	
Additional data availability information:	Tick here if your circumstances are not covered by the questions above and you need the journal’s help to make your data available.

Powered by Editorial Manager® and ProduXion Manager® from Aries Systems Corporation

Manuscript Click here to access/download;Manuscript;Revised MS.docx

Cognitive Costs and Gait Parameters During Single- and Dual-Task Conditions: A Comparative Study in Individuals With and Without Non-Specific Neck Pain

*Ibrahim M. Moustafa[1, 2] * , Shorouk Abu-Ghosh[1], Amal Ahbouch[1, 2] Shima Abdollah Mohammad Zadeh[1,2] ,Meeyoung Kim[1,2], Iman Khowailed [1]*

1 Department of Physiotherapy, College of Health Sciences, University of Sharjah, Sharjah 27272, United Arab Emirates

2 Neuromusculoskeletal Rehabilitation Research Group, RIMHS–Research Institute of Medical and Health Sciences, University of Sharjah, Sharjah 27272, United Arab Emirates

*Corresponding author
Email : Iabuamr@sharjah.ac.ae (IMM)

Abstract:

Background: The high incidence of non-specific neck pain (NSNP) among students, commonly associated with prolonged sedentary behavior and extensive use of electronic devices, highlights the need to assess its impact on cognitive and motor functions. This study aims to evaluate the effects of NSNP on dual-task performance by analyzing gait parameters and cognitive performance during both single-task and dual-task conditions.

Methods: Forty-five participants with NSNP and forty-five age-matched controls were assessed using an optical motion-capture system. Participants underwent gait assessments during both single-task (without cognitive load) and dual-task conditions, in which the cognitive tasks involved simple mathematical computations.

Results: Results revealed that, under single-task conditions, differences in gait parameters between groups were not statistically significant. However, under dual-task conditions, participants with NSNP exhibited significant impairments in gait parameters and higher cognitive costs ($p < 0.05$). Correlation analysis indicated that pain intensity was significantly associated with cognitive cost and gait parameter alterations during dual-task conditions ($p < 0.05$).

Conclusions: These findings suggest that NSNP significantly elevates the cognitive effort required during dual tasking. These finding emphasizes on the need for interventions to alleviate neck pain and improve both physical and cognitive health.

Keywords: Non-specific neck pain (NSNP); dual-task performance; cognitive load; gait parameters; pain intensity; cognitive cost

1. Introduction

Neck pain has become increasingly prevalent among students, mainly due to prolonged periods of studying, sedentary behavior, and extensive use of electronic devices [1]. As students spend more time hunched over books and screens, their necks bear the burden of the strain [2]. This non-specific neck pain (NSNP), which is not linked to any specific pathology or anatomical defect, affects a significant number of students and can lead to considerable discomfort, impacting not only their academic performance but also their overall quality of life [3].

The prevalence of neck pain among students highlights the importance of understanding its causes and effects. Factors such as poor posture, lack of ergonomic workstations, and inadequate physical activity contribute to the development of this condition [4]. Evidence has shown that improper use of pillows, prolonged use of electronic devices, and poor sitting posture are significant risk factors for neck pain in this population [5]. Moreover, neck pain is influenced by various modifiable and non-modifiable risk factors, such as advanced age, female gender, low social support, and a history of neck or lower back pain, emphasizing the need for prevention and early diagnosis [6].

It is crucial for students to take proactive steps to alleviate neck pain, such as practicing proper posture, taking regular breaks, incorporating neck stretches into their daily routines, and seeking medical attention if necessary [7]. By addressing neck pain and prioritizing their well-being, students can enhance their focus, productivity, and overall health[8,9]. Educational institutions can also play a role by promoting ergonomic practices and providing resources for physical well-being [10].

While the physical symptoms of NSNP are well-documented, there is a significant and noteworthy surge in interest surrounding the comprehensive understanding of its cognitive implications. It is undeniably established that chronic pain, including neck pain, can profoundly and unequivocally influence cognitive function through an intricate network of mechanisms[11]. These mechanisms encompass pain-induced distraction, extensively altered sensory input, and the overwhelming psychological stress that is inherently intertwined with the chronically discomforting nature of this condition [12]. These multifaceted and interrelated factors collectively and synergistically impose a substantial cognitive burden upon individuals, rendering the performance of tasks that necessitate concurrent physical and cognitive effort all the more arduous and formidable [13].

Literature has shown that chronic pain conditions can impair cognitive performance, particularly in tasks requiring attention, memory, and executive function [14]. However, it remains to be seen how mild to moderate neck pain among students affects their gait parameters, especially under dual-task conditions. The dual-task paradigm, which involves performing a cognitive task while simultaneously engaging in a physical activity, is particularly useful for studying the influence of cognitive load on motor performance [15].

In this section, it would be better to bring research related to neck pain on students or other groups.

Understanding dual-task performance can provide essentials insights into the impact of neck pain on individuals' ability to effectively manage multiple concurrent tasks [16]. This is particularly crucial for various daily activities where cognitive-motor integration is essential [17]. For instance, students frequently find themselves needing to engage in conversations while walking or engaging in mental calculations while in motion. These situations necessitate seamless coordination between cognitive and motor functions [18]. However, for individuals experiencing neck pain, the additional burden of discomfort and pain may intensify the challenges associated with maintaining equilibrium and coordination, potentially resulting in compromised performance for both tasks at hand [19].

Therefore, it is crucial to understand how neck pain affects dual-task performance to fully grasp the overall impact of this condition. Additionally, by exploring the interplay between physical discomfort and cognitive load, we can better appreciate the multifaceted nature of neck pain and its broader implications. The purpose of this study is to investigate the effects of NSNP on dual-task performance by examining gait parameters and cognitive performance during single-task and dual-task conditions among students. By comparing individuals with and without neck pain, we seek to identify the cognitive costs associated with managing dual tasks in the presence of neck pain. Ultimately, our goal is to promote a holistic approach to student well-being, recognizing the interconnectedness of physical and mental health in achieving optimal academic and personal outcomes.

2. Materials and Methods

2.1. Study Design, Participants and Setting

This comparative study was designed to assess the impact of cognitive dual-tasking on gait parameters in students experiencing from NSNP, in comparison to a well-matched control group without neck pain. The research was conducted in the laboratories of the University of Sharjah, with subjects recruited from among the university's students and staff through social media outreach. Recruitment took place from January to May 2024.

The title of your work is related to students with neck pain, but in this section you mentioned the statistical population as students and university staff, and this is a big problem because it needs to be standardized.

2.2. Ethical Considerations

The study received ethical approval from the University of Sharjah's College of Health Sciences (Ethical approval number: REC-23-05-11-03-S). All participants provided their informed written consent prior to the start of data collection. The study adhered to established ethical guidelines and regulations

Wouldn't it be better to exclude training history from the study?

2.3. Inclusion and exclusion criteria:

- Participants were eligible for the NSNP group if they met the following criteria:

1. Age between 18 and 24 years;
2. Experience of NSNP for at least three months, with a pain intensity rating between 30 and 70 on the Visual Analog Scale (VAS);
3. Affiliation as a student at the University of Sharjah;
4. Willingness to participate in the study and provide informed consent.

The control group consisted of individuals within the same age range and affiliation who reported no neck pain.

- Participants were excluded from the study if they had:

1. Previous fractures or operations on the neck or shoulders;
2. Previous major injuries or surgical procedures involving the musculoskeletal system;
3. Diagnosis of fibromyalgia, cervical radiculopathy/myelopathy, or cognitive impairments;
4. Deformities in the spine or limbs.

These criteria ensured a homogeneous sample, reducing potential confounding factors that could influence gait or cognitive performance outcomes.

2.3. Study Tools and Outcome Measures

In the entry criteria section, you mentioned pain intensity based on the VAS scale, but the tool you are using is the Numerical Pain Rating Scale. It would be better to also include pain intensity in that section based on the tool you are using.

2.3.1. Numerical Pain Rating Scale

Participants quantified their neck pain intensity using a numerical pain rating scale, a validated and reliable instrument for measuring pain intensity.

2.3.2. BTS GAITLAB System

The spatiotemporal and kinematic parameters of gait were assessed using an optical motion-capture system consisting of 8 infrared cameras (Smart-D, BTS Bioengineering, Milan, Italy) running at a frequency of 120 Hz. Anthropometric measurements, such as height, weight, and various body segment widths, were obtained before the experimental tests. Spherical reflective passive markers were placed on the subjects' skin following the protocol outlined by Davis et al.

2.3.3. Dual Task Cost (DTC) Percentage

The analysis was based on average performance from three dual-task trials to establish a comprehensive measure of the impact of cognitive load on gait. To calculate the Dual Task Cost (DTC) percentage, the disparity between single and dual-task performance scores was determined, and then divided by the single-task performance score before being multiplied by 100. For instance, if the average speed during single-task walking was 1.2 m/s and during dual-task walking was 1.0 m/s, the DTC percentage would be calculated as $((1.2 - 1.0) / 1.2) \times 100 = 16.67\%$.

2.3.4. Spatiotemporal Parameters

The spatiotemporal parameters of the participants, including step length, speed, and cadence, were measured under both single-task and dual-task conditions using the BTS gait lab system

2.4. Study Procedure

Participants were asked to walk at their chosen pace along a 10-meter walkway, with their 3D marker trajectories recorded by cameras under two different scenarios: single-task and dual-task. In the single-task scenario, participants walked at their chosen speed without performing any additional tasks, completing three trials to establish their baseline gait characteristics. In the dual-task scenario, participants walked while engaging in various cognitive tasks, such as answering yes or no questions, counting, or performing simple math calculations. Each participant completed a minimum of three trials for each scenario. Upon completion of the tests, the raw data were analyzed using specialized software (Smart Analyzer, BTS Bioengineering, Milan, Italy) to derive the necessary parameters.

2.5. Sample Size Determination

It is best to determine the sample size based on the severity of pain, which is the outcome measure of your study. Review related articles.

The sample size was determined based on an effect size of 0.5, a two-tailed significance level (α) of 0.05, and a statistical power ($1-\beta$) of 0.80. Using G*Power 3.1 software, the calculation indicated that a minimum of 40 participants per group (neck pain and no neck pain) would be required to detect a significant difference with 80% power at a 5% significance level. To accommodate potential dropouts, 5 additional participants were included per group, bringing the total to 45 participants per group.

2.6. Statistical Analysis

To assess the normal distribution of all baseline descriptive variables, the Kolmogorov-Smirnov test was utilized. Continuous variables are presented as mean values alongside their standard deviations (SD). Levene's test was applied at a 95% confidence interval to examine the homogeneity of variances, with significance defined as p-values less than 0.05. Descriptive statistics (mean ± SD) were reported for each respective time point. To confirm group equivalence, chi-squared tests were applied to categorical variables, while Student's t-tests were used for continuous variables. The comparison of mean values between groups was conducted using the Student's t-test, with statistical significance established at a p-value of less than 0.05. Effect size was assessed using Cohen's d, where d values around 0.2, 0.5, and 0.8 corresponded to negligible, moderate, and substantial clinical significance, respectively. Pearson's correlation coefficients (r) were utilized to investigate the associations between neck pain severity and spatiotemporal gait parameters in both single and dual-task conditions, as well as the relationship between neck pain severity and cognitive cost. All analyses were performed using SPSS version 20.0 (SPSS Inc., Chicago, IL, USA), ensuring the assumptions of normality and homogeneity of variance were met prior to analysis.

3. Results

3.1. Demographics

Over 200 potential participants were initially screened, with the most common exclusion criterion being the pain levels outside 30-70 on the VAS or having neck pain for less than three months. Ultimately, 45 participants with NSNP (25 males, 20 females) and 45 matched controls (based on age, BMI, and sex) without neck pain were recruited for the study ($p>0.05$). The significant difference in VAS validates the classification of the groups based on neck pain status. The demographic characteristics and clinical variables of these participants are detailed in Table 1.

Table 1. Baseline demographic characteristics and clinical variables.

Variable	Neck Pain (n=45)	No Neck Pain (n=45)	p-value	Cohen's d
Age (years)	20.50 ± 2.0	20.00 ± 3.0	0.30	0.67
BMI	21.50 ± 1.7	21.40 ± 1.8	0.60	0.60

Gender (% Male)	55.5	55.5	1.00	< 0.001
Gender (% Female)	44.5	44.5	1.00	< 0.001
VAS	55±8	0		

3.2. Single Task Detailed Results

Table 2 presents the mean differences in spatiotemporal parameters between the neck pain and no neck pain groups during the single-task condition. Most variables exhibit non-significant differences. Most spatiotemporal parameters during the single-task condition do not show significant differences between the neck pain and no neck pain groups. However, significant differences were observed in swing time, mean velocity and step length, indicating an impact of neck pain on these specific parameters.

Table 2. Mean Differences between Spatiotemporal Parameters in Neck Pain and No Neck Pain Groups during Single Task.

Variable	Group	Mean ± SD	95% CI	Cohen's d	p-value
Stride time (s)	Neck Pain	1.25 ± 0.10	[1.22, 1.28]	0.20	0.15
	No Neck Pain	1.23 ± 0.10	[1.20, 1.26]		
Stance time (s)	Neck Pain	0.75 ± 0.05	[0.73, 0.77]	0.25	0.10
	No Neck Pain	0.74 ± 0.05	[0.72, 0.76]		
Swing time (s)	Neck Pain	0.45 ± 0.05	[0.43, 0.47]	0.60	< 0.001
	No Neck Pain	0.42 ± 0.05	[0.40, 0.44]		
Stance phase (%)	Neck Pain	62.0 ± 2.0	[61.3, 62.7]	0.30	0.05
	No Neck Pain	61.5 ± 2.0	[60.8, 62.2]		
Double support phase (%)	Neck Pain	12.0 ± 1.5	[11.5, 12.5]	0.15	0.20
	No Neck Pain	11.8 ± 1.5	[11.3, 12.3]		
Mean velocity m/s	Neck Pain	1.10 ± 0.10	[1.07, 1.13]	0.40	0.02
	No Neck Pain	1.15 ± 0.10	[1.12, 1.18]		
Mean velocity height (%height/s)	Neck Pain	70.0 ± 5.0	[68.5, 71.5]	0.10	0.50
	No Neck Pain	69.8 ± 5.0	[68.3, 71.3]		
Cadence (steps/min)	Neck Pain	110.0 ± 5.0	[108.5, 111.5]	0.20	0.15
	No Neck Pain	109.0 ± 5.0	[107.5, 110.5]		
Stride length (%height)	Neck Pain	75.0 ± 5.0	[73.5, 76.5]	0.15	0.20
	No Neck Pain	74.5 ± 5.0	[73.0, 76.0]		
Step length (m)	Neck Pain	0.60 ± 0.05	[0.58, 0.62]	0.60	< 0.001
	No Neck Pain	0.57 ± 0.05	[0.55, 0.59]		
Step width avg (m)	Neck Pain	0.09 ± 0.03	[0.08, 0.10]	0.10	0.50
	No Neck Pain	0.09 ± 0.03	[0.08, 0.10]		

3.3. Dual Task Detailed Results

Table 3 illustrates the mean differences in spatiotemporal parameters between the neck pain and no neck pain groups during the dual-task condition. Significant differences were observed in all

spatiotemporal parameters, except for the cadence, between the neck pain and no neck pain groups during the dual-task condition. This indicates that neck pain significantly impacts gait parameters under increased cognitive load.

Table 3. Mean Differences in Spatiotemporal Parameters Between Neck Pain and No Neck Pain Groups During Dual-Task Conditions.

Variable	Group	Mean ± SD	95% CI	Cohen's d	p-value
Stride time (s)	Neck Pain	1.50 ± 0.15	[1.45, 1.55]	0.80	< 0.001
	No Neck Pain	1.20 ± 0.10	[1.17, 1.23]		
Stance time (s)	Neck Pain	0.80 ± 0.05	[0.78, 0.82]	1.14	< 0.001
	No Neck Pain	0.70 ± 0.05	[0.68, 0.72]		
Swing time (s)	Neck Pain	0.50 ± 0.05	[0.48, 0.52]	0.80	< 0.001
	No Neck Pain	0.45 ± 0.05	[0.43, 0.47]		
Stance phase (%)	Neck Pain	65.0 ± 3.0	[64.1, 65.9]	0.80	0.01
	No Neck Pain	61.0 ± 2.5	[60.3, 61.7]		
Double support phase (%)	Neck Pain	13.0 ± 2.0	[12.4, 13.6]	0.80	< 0.001
	No Neck Pain	11.0 ± 1.5	[10.5, 11.5]		
Mean velocity m/s	Neck Pain	1.10 ± 0.10	[1.07, 1.13]	0.80	< 0.001
	No Neck Pain	1.20 ± 0.10	[1.17, 1.23]		
Mean velocity height (%height/s)	Neck Pain	65.0 ± 5.0	[63.5, 66.5]	0.80	< 0.001
	No Neck Pain	72.0 ± 5.0	[70.5, 73.5]		
Cadence (steps/min)	Neck Pain	110.0 ± 5.0	[108.5, 111.5]	0.60	0.30
	No Neck Pain	105.0 ± 5.0	[103.5, 106.5]		
Stride length (%height)	Neck Pain	75.0 ± 5.0	[73.5, 76.5]	1.00	< 0.001
	No Neck Pain	80.0 ± 5.0	[78.5, 81.5]		
Step length (m)	Neck Pain	0.60 ± 0.05	[0.58, 0.62]	1.10	< 0.001
	No Neck Pain	0.65 ± 0.05	[0.63, 0.67]		
Step width avg (m)	Neck Pain	0.09 ± 0.03	[0.08, 0.10]	0.60	< 0.001
	No Neck Pain	0.07 ± 0.02	[0.06, 0.08]		

3.4. Cognitive Cost Detailed Results

The cognitive cost comparison results, presented in Table 4, show significant differences across multiple gait parameters in the neck pain group**.** The Mean Difference in Cognitive Cost (CC) between Neck Pain and No Neck Pain. The cognitive cost values indicate that individuals with neck pain incur significantly higher cognitive costs across various gait parameters during dual-task conditions compared to those without neck pain. This finding suggests that neck pain exacerbates the cognitive effort required to maintain gait.

Table 4. Mean Differences in cognitive cost Between Neck Pain and No Neck Pain groups.

Variable	Group	Mean ± SD	95% CI	Cohen's d	p-value
CC Stride time	Neck Pain	25.79 ± 5.95	[23.99, 27.59]	0.87	< 0.001

	No Neck Pain	5.19 ± 3.50	[4.19, 6.19]		
CC Stance time	Neck Pain	8.43 ± 4.00	[7.43, 9.43]	0.89	< 0.001
	No Neck Pain	-6.75 ± 3.75	[-7.75, -5.75]		
CC Swing time	Neck Pain	2.78 ± 3.00	[2.08, 3.48]	-0.72	< 0.001
	No Neck Pain	15.03 ± 4.68	[13.53, 16.53]		
CC Stance phase	Neck Pain	8.49 ± 3.19	[7.49, 9.49]	0.64	0.01
	No Neck Pain	1.40 ± 3.00	[0.70, 2.10]		
CC Double support phase	Neck Pain	19.87 ± 5.69	[18.07, 21.67]	0.66	0.01
	No Neck Pain	1.11 ± 5.00	[-0.39, 2.61]		
CC Mean velocity	Neck Pain	12.36 ± 6.45	[10.36, 14.36]	-0.75	0.02
	No Neck Pain	6.85 ± 4.00	[5.85, 7.85]		
CC Mean velocity height	Neck Pain	13.34 ± 4.90	[11.84, 14.84]	-0.80	< 0.001
	No Neck Pain	3.18 ± 4.00	[2.18, 4.18]		
CC Cadence	Neck Pain	4.49 ± 4.00	[3.49, 5.49]	-0.30	< 0.001
	No Neck Pain	1.46 ± 4.00	[0.46, 2.46]		
CC Stride length	Neck Pain	6.84 ± 4.00	[5.84, 7.84]	-0.81	0.01
	No Neck Pain	2.41 ± 4.00	[1.41, 3.41]		
CC Step length	Neck Pain	9.98 ± 5.00	[8.48, 11.48]	-1.13	0.01
	No Neck Pain	4.39 ± 4.00	[3.39, 5.39]		
CC Step width avg	Neck Pain	12.43 ± 4.90	[10.93, 13.93]	0.49	0.01
	No Neck Pain	9.91 ± 5.00	[8.41, 11.41]		

3.5. Correlational Results

The single-task condition reveals no significant correlations between pain intensity and gait parameters ($p > 0.05$). This indicates that pain intensity does not significantly influence performance parameters during single tasks in either group.

The correlation table for the dual-task condition illustrates significant correlations between pain intensity and various performance parameters, suggesting that pain intensity substantially influences gait parameters during dual-task activities (Table 5).

Table 5. Correlation Between Pain Intensity and Gait Parameters During Dual-Task Conditions.

Parameter	Spearman r	p-value
Stride time (s)	-0.68	< 0.001
Stance Time (s)	-0.56	< 0.001
Swing Time (s)	0.31	< 0.001
Stance Phase (%)	-0.56	< 0.001
Swing Phase (%)	0.53	< 0.001
Single Support Phase (%)	0.40	< 0.001
Double Support Phase (%)	-0.55	< 0.001
Mean Velocity (m/s)	0.28	< 0.001
Mean Velocity (%height/s)	0.49	< 0.001
Cadence (steps/min)	0.20	< 0.001
Stride Length (%height)	0.39	< 0.001

Step Length (m)	0.60	< 0.001
Step Width (m)	-0.40	< 0.001

The correlation table for cognitive cost values illustrates a connection between pain intensity and cognitive cost measures (Table 6). The results suggest that pain intensity significantly influences the cognitive cost related to various gait parameters, indicating that as pain intensity rises, the cognitive effort needed to sustain gait during dual-task scenarios also increases.

Table 6. Correlation Between Pain Intensity and Cognitive Cost.

Parameter	Spearman r	p-value
CC Stride time (s)	-0.61	< 0.001
CC Stance Time (s)	-0.41	< 0.001
CC Swing Time (s)	-0.35	< 0.001
CC Stance Phase (%)	-0.35	< 0.001
CC Swing Phase (%)	-0.45	< 0.001
CC Single Support Phase (%)	-0.23	0.04
CC Double Support Phase (%)	-0.21	0.04
CC Mean Velocity (m/s)	-0.37	0.01
CC Mean Velocity (%height/s)	-0.30	0.01
CC Cadence (steps/min)	-0.22	0.01
CC Stride Length (%height)	-0.32	0.01
CC Step Length (m)	-0.55	< 0.001
CC Step Width (m)	-0.50	< 0.001

CC: Cognitive cost

4. Discussion

Explain this section more and better because the superiority of your research over previous studies should be more prominent in the discussion section.

Our study showed that how NSNP influences cognitive performance during dual-task activities, bringing forth crucial findings that deepen our understanding of the connection between musculoskeletal discomfort and cognitive function. The data demonstrate that individuals with NSNP incur higher cognitive costs when performing dual tasks compared to those without neck pain, indicating that NSNP might elevate cognitive demands during gait-related activities. These outcomes are in line with prior research, which has shown that neck pain can lead to both physiological and neurological disruptions, thereby affecting physical and cognitive functions. [20].

These findings enhance our grasping the intricate nature of neck pain and its wider implications. This observation aligns with cognitive load theory, which argues that physical discomfort and proprioceptive errors linked to neck pain can raise cognitive load, thereby negatively affecting cognitive performance. Recent studies reinforce this idea by showing that neck pain disrupts sensorimotor control and neural processing, leading to an increased cognitive burden and reduced efficiency in cognitive tasks.[21]

4.1. Interpretation of Findings

The discrepancies in dual-task performance between the neck pain group and the control group are in line with prior studies that indicate musculoskeletal discomfort can influence cognitive processes [22–24]. The increased cognitive load observed in the neck pain group may result from disrupted sensory input and proprioception caused by pain and discomfort [25–27]. This phenomenon is like phantom limb pain, which emerges from maladaptive cortical reorganization after sensory input loss, suggesting that NSNP might also trigger maladaptive changes in the sensory and motor cortices [28,29]. This is further supported by research showing that both conditions involve reorganization within the primary somatosensory and motor cortices, which contributes to chronic pain [30,31]. These disturbances may impose an increased cognitive load as the brain compensates for compromised

postural stability. It is a phenomenon elucidated by prior research which underscores deficits in attention, processing speed, and memory among individuals experiencing musculoskeletal pain. [32].

Several mechanisms may account for the increased cognitive burden observed in the NSNP group. First, neck pain creates biomechanical stress on the neck and shoulders, leading to muscle fatigue and discomfort, which can reduce the cognitive resources available for task execution [6]. Additionally, neck pain is linked to altered proprioceptive function, which can disrupt sensory integration and further increase the cognitive load needed to maintain balance and perform tasks [33]. Structural changes in the cervical spine associated with neck pain may also impair cerebral blood flow, affecting cognitive functions such as attention and memory [34,35]. Deafferentation due to poor posture can intensify these cognitive deficits, as research shows that the loss of sensory nerve signals from poor posture increases cognitive load by disrupting proprioceptive feedback and amplifying the brain's compensatory efforts [36,37].

Additionally, neuroplasticity plays a pivotal role in the interplay between cognitive processes and musculoskeletal discomfort [22]. Neuroplastic changes induced by altered sensory input from neck pain can result in both beneficial and adverse cognitive outcomes [38]. Studies indicate that neuroplastic mechanisms, such as synaptogenesis and cortical reorganization, are engaged to accommodate the elevated cognitive demands imposed by neck pain. These processes highlight the brain's profound capacity to reorganize and recalibrate its neural networks in response to altered sensory inputs, thereby preserving cognitive function despite the presence of physical impairments [38,39].

4.2. Implications for Clinical Practice

The robust correlations between pain intensity and cognitive dual-task performance emphasize the necessity for comprehensive assessment protocols that address both musculoskeletal and cognitive dimensions. Clinicians should recognize the potential cognitive effects of neck pain and incorporate strategies to manage musculoskeletal discomfort within their rehabilitation approaches. [40]. This is particularly crucial for populations at higher risk of cognitive decline, including older adults and individuals with neurological disorders [41,42]. Our research also highlights the importance of dual-task gait assessments as a crucial tool for identifying cognitive impairments. By integrating cognitive and motor tasks, these assessments provide a more realistic measure of everyday functioning, making them essential for detecting subtle cognitive deficits that may not be evident in single-task evaluations. [43]. These findings have significant implications for early intervention and continuous monitoring of cognitive health in clinical practice. Clinicians should incorporate neck pain assessments into routine evaluations, especially for individuals exhibiting cognitive concerns. [44]. Embracing interdisciplinary approaches involving physical therapists, occupational therapists, and cognitive specialists can provide comprehensive care that addresses both the physical and cognitive challenges associated with neck pain [45]. Early intervention and patient education emphasizing the importance of proper posture and effective management of musculoskeletal discomfort are essential for mitigating the risk of developing neck pain and its consequent cognitive impairments [46].

4.3. ***Practical Applications***

People suffering from neck pain are more likely to experience increased cognitive fatigue and a decline in efficiency when performing tasks that require both mental and physical effort simultaneously [47]. This can greatly impact everyday activities, work productivity, and overall quality of life [48]. Additionally, chronic neck pain can lead to enduring health problems, such as persistent pain, headaches, and even potential cognitive decline over time [49–51].

To counteract the negative effects of neck pain, it is vital to implement strategies that alleviate discomfort. Such strategies might include physical therapy, workplace ergonomic modifications, and exercises designed to strengthen the neck and upper back muscles [52]. Furthermore, taking regular

breaks and performing posture checks during extended computer use can help maintain proper alignment and reduce the risk of developing neck pain [53].

Limitations and Future Directions

Although our study is well-designed and yields significant findings, it is important to recognize certain limitations. The cross-sectional design restricts our ability to infer causality, emphasizing the need for longitudinal studies to better understand the temporal relationship between neck pain and cognitive decline. Additionally, our sample consisted primarily of university students and staff, which may not fully represent the broader population. Future studies should aim to include more diverse participant groups to improve the generalizability of the results.

Further research is necessary to explore the mechanisms connecting musculoskeletal discomfort with cognitive function. Specifically, examining the roles and connectivity of different brain regions in individuals experiencing musculoskeletal pain could shed light on the neurophysiological underpinnings of our findings. Moreover, intervention studies that investigate the impact of pain relief on cognitive outcomes will be critical for evaluating the effectiveness of targeted treatments.

5. Conclusions

Our research indicates that NSNP markedly raises the cognitive demands of walking, emphasizing the urgent need to manage musculoskeletal discomfort to safeguard cognitive function. Implementing targeted strategies to alleviate neck pain could lead to improvements in both physical and mental health, thereby enhancing overall quality of life and daily activities. Additional studies are necessary to investigate the long-term consequences of neck pain and to determine the effectiveness of different interventions in minimizing its cognitive effects.

Funding: This research did not receive any specific grant from funding agencies in the public, commercial, or not-for-profit sectors.

Data Availability Statement: Data is provided as supplementary information file.

Conflicts of Interest: The authors declare no conflicts of interest.

References

1. Mahdavi, S.B.; Mazaheri-Tehrani, S.; Riahi, R.; Vahdatpour, B.; Kelishadi, R. Sedentary Behavior and Neck Pain in Children and Adolescents; a Systematic Review and Meta-Analysis. Health Promot Perspect 2022, 12, 240, doi:10.34172/HPP.2022.31.
2. Sigedar, P.; Giri, P. Text Neck Syndrome: An Emerging Epidemic during COVID-19 Pandemic. New Horizons in Medicine and Medical Research Vol. 11 2022, 1–7, doi:10.9734/BPI/NHMMR/V11/2640B.
3. Tariq, F.; Akhtar, M.W.; Mahmood, M.; Saeed, S.; Burhan, M.; Shafqat, A.; Munawar, A. Frequency of Neck Pain and Associated Disability in Medical Students. Journal of Health and Rehabilitation Research 2024, 4, 1647–1651, doi:10.61919/JHRR.V4I1.658.
4. Gheysvandi, E.; Dianat, I.; Heidarimoghadam, R.; Tapak, L.; Karimi-Shahanjarini, A.; Rezapur-Shahkolai, F. Neck and Shoulder Pain among Elementary School Students: Prevalence and Its Risk Factors. BMC Public Health 2019, 19, 1–11, doi:10.1186/S12889-019-7706-0/TABLES/6.
5. Gao, Y.; Chen, Z.; Chen, S.; Wang, S.; Lin, J. Risk Factors for Neck Pain in College Students: A Systematic Review and Meta-Analysis. BMC Public Health 2023, 23, 1–13, doi:10.1186/S12889-023-16212-7/.
6. Kazeminasab, S.; Nejadghaderi, S.A.; Amiri, P.; Pourfathi, H.; Araj-Khodaei, M.; Sullman, M.J.M.; Kolahi, A.A.; Safiri, S. Neck Pain: Global Epidemiology, Trends and Risk Factors. BMC Musculoskelet Disord 2022, 23, 1–13, doi:10.1186/S12891-021-04957-4.
7. Yang, S.; Boudier-Revéret, M.; Yi, Y.G.; Hong, K.Y.; Chang, M.C.; Yang, S.; Boudier-Revéret, M.; Yi, Y.G.; Hong, K.Y.; Chang, M.C. Treatment of Chronic Neck Pain in Patients with Forward Head Posture: A Systematic Narrative Review. Healthcare 2023, Vol. 11, Page 2604 2023, 11, 2604, doi:10.3390/HEALTHCARE11192604.
8. Bertozzi, L.; Gardenghi, I.; Turoni, F.; Villafañe, J.H.; Capra, F.; Guccione, A.A.; Pillastrini, P. Effect of Therapeutic Exercise on Pain and Disability in the Management of Chronic Nonspecific Neck Pain: Systematic Review and Meta-Analysis of Randomized Trials. Phys Ther 2013, 93, 1026–1036, doi:10.2522/PTJ.20120412.
9. Johnston, V.; Chen, X.; Welch, A.; Sjøgaard, G.; Comans, T.A.; McStea, M.; Straker, L.; Melloh, M.; Pereira, M.; O'Leary, S. A Cluster-Randomized Trial of Workplace Ergonomics and Neck-Specific Exercise versus Ergonomics and Health Promotion for Office Workers to Manage Neck Pain – a Secondary Outcome Analysis. BMC Musculoskelet Disord 2021, 22, 1–12, doi:10.1186/S12891-021-03945-Y.
10. Cruz, M.J.J.; Piñon, I.S.V.D.; Janice, M.; Gumasing, J.; Samantha, I.; Dela Cruz, V.; Piñon, A.A.; Nicolaison, H.; Rebong, M.; Luis, D.; et al. Ergonomic Factors Affecting the Learning Motivation and Academic Attention of SHS Students in Distance Learning. Sustainability 2023, Vol. 15, Page 9202 2023, 15, 9202, doi:10.3390/SU15129202.
11. Shahidi, B.; Curran-Everett, D.; Maluf, K.S. Psychosocial, Physical, and Neurophysiological Risk Factors for Chronic Neck Pain: A Prospective Inception Cohort Study. J Pain 2015, 16, 1288–1299, doi:10.1016/J.JPAIN.2015.09.002.
12. Wippert, P.M.; Wiebking, C. Stress and Alterations in the Pain Matrix: A Biopsychosocial Perspective on Back Pain and Its Prevention and Treatment. Int J Environ Res Public Health 2018, 15, doi:10.3390/IJERPH15040785.
13. Barnhart, W.R.; Buelow, M.T.; Trost, Z. Effects of Acute Pain and Pain-Related Fear on Risky Decision-Making and Effort during Cognitive Tests. J Clin Exp Neuropsychol 2019, 41, 1033–1047, doi:10.1080/13803395.2019.1646711.
14. Morogiello, J.; Murray, N.G.; Hunt, T.N.; Harris, B.S.; Szekely, B.J.; Shaver, G.W. The Effect of Acute Pain on Executive Function. J Clin Transl Res 2019, 4, 113, doi:10.18053/jctres.04.201802.003.
15. Broglio, S.P.; Tomporowski, P.D.; Ferrara, M.S. Balance Performance with a Cognitive Task: A Dual-Task Testing Paradigm. Med Sci Sports Exerc 2005, 37, 689–695, doi:10.1249/01.MSS.0000159019.14919.09.

16. Saadat, M.; Shaterzadeh, M.J.; Negahban, H.; Arastoo, A.; Zahednegad, S. The Effect of Dual-Tasking on 'Timed up and Go' Performance in Subjects with Chronic Nonspecific Neck Pain. Biomedical and Pharmacology Journal 2015, 8, 789–794, doi:10.13005/BPJ/784.
17. Schaefer, S.; Schumacher, V. The Interplay between Cognitive and Motor Functioning in Healthy Older Adults: Findings from Dual-Task Studies and Suggestions for Intervention. Gerontology 2011, 57, 239–246, doi:10.1159/000322197.
18. McPhee, A.M.; Cheung, T.C.K.; Schmuckler, M.A. Dual-Task Interference as a Function of Varying Motor and Cognitive Demands. Front Psychol 2022, 13, 952245, doi:10.3389/FPSYG.2022.952245/BIBTEX.
19. Sremakaew, M.; Sungkarat, S.; Treleaven, J.; Uthaikhup, S. Effects of Tandem Walk and Cognitive and Motor Dual- Tasks on Gait Speed in Individuals with Chronic Idiopathic Neck Pain: A Preliminary Study. Physiother Theory Pract 2021, 37, 1210–1216, doi:10.1080/09593985.2019.1686794.
20. Qu, N.; Tian, H.C.; De Martino, E.; Zhang, B. Neck Pain: Do We Know Enough About the Sensorimotor Control System? Front Comput Neurosci 2022, 16, doi:10.3389/FNCOM.2022.946514.
21. de Jong, T. Cognitive Load Theory, Educational Research, and Instructional Design: Some Food for Thought. Instr Sci 2010, 38, 105–134, doi:10.1007/S11251-009-9110-0/METRICS.
22. Sui, S.X.; Balanta-Melo, J.; Pasco, J.A.; Plotkin, L.I. Musculoskeletal Deficits and Cognitive Impairment: Epidemiological Evidence and Biological Mechanisms. Curr Osteoporos Rep 2022, 20, 260, doi:10.1007/S11914-022-00736-9.
23. Khera, T.; Rangasamy, V. Cognition and Pain: A Review. Front Psychol 2021, 12, doi:10.3389/FPSYG.2021.673962.
24. Moriarty, O.; McGuire, B.E.; Finn, D.P. The Effect of Pain on Cognitive Function: A Review of Clinical and Preclinical Research. Prog Neurobiol 2011, 93, 385–404, doi:10.1016/J.PNEUROBIO.2011.01.002.
25. Treleaven, J.; Peterson, G.; Ludvigsson, M.L.; Kammerlind, A.S.; Peolsson, A. Balance, Dizziness and Proprioception in Patients with Chronic Whiplash Associated Disorders Complaining of Dizziness: A Prospective Randomized Study Comparing Three Exercise Programs. Man Ther 2016, 22, 122–130, doi:10.1016/J.MATH.2015.10.017.
26. Brumagne, S.; Cordo, P.; Verschueren, S. Proprioceptive Weighting Changes in Persons with Low Back Pain and Elderly Persons during Upright Standing. Neurosci Lett 2004, 366, 63–66, doi:10.1016/j.neulet.2004.05.013.
27. Treleaven, J. Sensorimotor Disturbances in Neck Disorders Affecting Postural Stability, Head and Eye Movement Control. Man Ther 2008, 13, 2–11, doi:10.1016/J.MATH.2007.06.003.
28. Borrell, J.A.; Manattu, A.K.; Copeland, C.; Fraser, K.; D'Ovidio, A.; Granatowicz, Z.; Lesiak, A.C.; Figy, S.C.; Zuniga, J.M. Phantom Limb Therapy Improves Cortical Efficiency of the Sensorimotor Network in a Targeted Muscle Reinnervation Amputee: A Case Report. Front Neurosci 2023, 17, 1130050, doi:10.3389/FNINS.2023.1130050/BIBTEX.
29. Flor, H.; Elbert, T.; Knecht, S.; Wienbruch, C.; Pantev, C.; Birbaumers, N.; Larbig, W.; Taub, E. Phantom-Limb Pain as a Perceptual Correlate of Cortical Reorganization Following Arm Amputation. Nature 1995, 375, 482–484, doi:10.1038/375482A0.
30. Flor, H.; Diers, M. Sensorimotor Training and Cortical Reorganization. NeuroRehabilitation 2009, 25, 19–27, doi:10.3233/NRE-2009-0496.
31. Tsao, H.; Danneels, L.A.; Hodges, P.W. ISSLS Prize Winner: Smudging the Motor Brain in Young Adults with Recurrent Low Back Pain. Spine (Phila Pa 1976) 2011, 36, 1721–1727, doi:10.1097/BRS.0B013E31821C4267.
32. Demurtas, J.; Schoene, D.; Torbahn, G.; Marengoni, A.; Grande, G.; Zou, L.; Petrovic, M.; Maggi, S.; Cesari, M.; Lamb, S.; et al. Physical Activity and Exercise in Mild Cognitive Impairment and Dementia: An Umbrella Review of Intervention and Observational Studies. J Am Med Dir Assoc 2020, 21, 1415-1422.e6, doi:10.1016/J.JAMDA.2020.08.031.
33. Peng, B.; Yang, L.; Li, Y.; Liu, T.; Liu, Y. Cervical Proprioception Impairment in Neck Pain-Pathophysiology, Clinical Evaluation, and Management: A Narrative Review. Pain and Therapy 2021 10:1 2021, 10, 143–164, doi:10.1007/S40122-020-00230-Z.

34. Zhao, R.; Su, Q.; Chen, Z.; Sun, H.; Liang, M.; Xue, Y. Neural Correlates of Cognitive Dysfunctions in Cervical Spondylotic Myelopathy Patients: A Resting-State FMRI Study. Front Neurol 2020, 11, doi:10.3389/FNEUR.2020.596795.
35. Kim, H.W.; Yong, H.; Shea, G.K.H. Blood-Spinal Cord Barrier Disruption in Degenerative Cervical Myelopathy. Fluids Barriers CNS 2023, 20, 1–18, doi:10.1186/S12987-023-00463-Y/TABLES/2.
36. Chen, Z.; Zhao, R.; Wang, Q.; Yu, C.; Li, F.; Liang, M.; Zong, Y.; Zhao, Y.; Xiong, W.; Su, Z.; et al. Functional Connectivity Changes of the Visual Cortex in the Cervical Spondylotic Myelopathy Patients: A Resting-State FMRI Study. Spine (Phila Pa 1976) 2020, 45, E272–E279, doi:10.1097/BRS.0000000000003245.
37. Sitsen, E.; Khalili-Mahani, N.; de Rover, M.; Dahan, A.; Niesters, M. Effect of Spinal Anesthesia-Induced Deafferentation on Pain Processing in Healthy Male Volunteers: A Task-Related FMRI Study. Frontiers in Pain Research 2022, 3, doi:10.3389/FPAIN.2022.1001148/FULL.
38. Pascoal-Faria, P.; Yalcin, N.; Fregni, F. Neural Markers of Neuropathic Pain Associated with Maladaptive Plasticity in Spinal Cord Injury. Pain Pract 2015, 15, 371–377, doi:10.1111/PAPR.12237.
39. Marzola, P.; Melzer, T.; Pavesi, E.; Gil-Mohapel, J.; Brocardo, P.S. Exploring the Role of Neuroplasticity in Development, Aging, and Neurodegeneration. Brain Sciences 2023, Vol. 13, Page 1610 2023, 13, 1610, doi:10.3390/BRAINSCI13121610.
40. Tanaka, K.; Nishigami, T.; Mibu, A.; Imai, R.; Manfuku, M.; Tanabe, A. Combination of Pain Location and Pain Duration Is Associated with Central Sensitization-Related Symptoms in Patients with Musculoskeletal Disorders: A Cross-Sectional Study. Pain Pract 2021, 21, 646–652, doi:10.1111/PAPR.13005.
41. Lipardo, D.S.; Tsang, W.W.N. Effects of Combined Physical and Cognitive Training on Fall Prevention and Risk Reduction in Older Persons with Mild Cognitive Impairment: A Randomized Controlled Study. Clin Rehabil 2020, 34, 773–782, doi:10.1177/0269215520918352.
42. Ramírez, F.; Gutiérrez, M. Dual-Task Gait as a Predictive Tool for Cognitive Impairment in Older Adults: A Systematic Review. Front Aging Neurosci 2021, 13, doi:10.3389/FNAGI.2021.769462.
43. Kannan, L.; Pitts, J.; Szturm, T.; Purohit, R.; Bhatt, T. Perturbation-Based Dual Task Assessment in Older Adults with Mild Cognitive Impairment. Frontiers in Rehabilitation Sciences 2024, 5, 1384582, doi:10.3389/FRESC.2024.1384582/BIBTEX.
44. Weigl, M.; Letzel, J.; Angst, F. Prognostic Factors for the Improvement of Pain and Disability Following Multidisciplinary Rehabilitation in Patients with Chronic Neck Pain. BMC Musculoskelet Disord 2021, 22, 1–11, doi:10.1186/S12891-021-04194-9/TABLES/5.
45. Engebretsen, K.B.; Taso, M.; Bjorland, S.; Jenssen, H.K.; Skaara, H.E.; Brox, J.I. A Functional Intervention within a Cognitive Approach to Chronic Cervical Radiculopathy. BMC Musculoskeletal Disorders 2024 25:1 2024, 25, 1–9, doi:10.1186/S12891-024-07743-0.
46. Maekawa, M.; Yoshizawa, E.; Hayata, G.; Ohashi, S. Physical and Psychological Effects of Postural Educational Intervention for Students Experienced School Refusal. Current Psychology 2023, 42, 3510–3519, doi:10.1007/S12144-021-01677-9.
47. Aitken, B.; MacMahon, C. Shared Demands Between Cognitive and Physical Tasks May Drive Negative Effects of Fatigue: A Focused Review. Front Sports Act Living 2019, 1, 45, doi:10.3389/FSPOR.2019.00045.
48. van As, S.; Beckers, D.G.J.; Geurts, S.A.E.; Kompier, M.A.J.; Husain, M.; Veling, H. The Impact of Cognitive and Physical Effort Exertion on Physical Effort Decisions: A Pilot Experiment. Front Psychol 2021, 12, 645037, doi:10.3389/FPSYG.2021.645037/BIBTEX.
49. Al-Khazali, H.M.; Krøll, L.S.; Ashina, H.; Melo-Carrillo, A.; Burstein, R.; Amin, F.M.; Ashina, S. Neck Pain and Headache: Pathophysiology, Treatments and Future Directions. Musculoskelet Sci Pract 2023, 66, 102804, doi:10.1016/J.MSKSP.2023.102804.
50. Anarte-Lazo, E.; Bernal-Utrera, C.; Montaño-Ocaña, J.; Falla, D.; Rodriguez-Blanco, C. Higher Neck Pain Intensity and the Presence of Psychosocial Factors Are More Likely When Headache Is Present after a Whiplash Injury: A Case-Control Study. Pain Medicine 2022, 23, 1529–1535, doi:10.1093/PM/PNAC038.

51. Dong, H. Multisite Chronic Pain: A Risk Factor for Dementia. Nature Reviews Neurology 2023 19:6 2023, 19, 331–332, doi:10.1038/s41582-023-00807-0.
52. Chen, X.; Coombes, B.K.; Sjøgaard, G.; Jun, D.; O'Leary, S.; Johnston, V. Workplace-Based Interventions for Neck Pain in Office Workers: Systematic Review and Meta-Analysis. Phys Ther 2018, 98, 40–62, doi:10.1093/PTJ/PZX101.
53. Alshehre, Y.M.; Pakkir Mohamed, S.H.; Nambi, G.; Almutairi, S.M.; Alharazi, A.A. Effectiveness of Physical Exercise on Pain, Disability, Job Stress, and Quality of Life in Office Workers with Chronic Non-Specific Neck Pain: A Randomized Controlled Trial. Healthcare 2023, Vol. 11, Page 2286 2023, 11, 2286, doi:10.3390/HEALTHCARE11162286.
