# Supplementary material for: Cognitive costs and gait parameters during single- and dual-task conditions: A comparative study in individuals with and without non-specific neck pain
Source: PLoS One. 2025 May 20;20(5):e0318963. doi: 10.1371/journal.pone.0318963 (PMC12091795; doi:10.1371/journal.pone.0318963)
Supplement: Attachment — Submitted filename: response.docx [file pone.0318963.s004.docx]

We would like to thank you for your time and effort in reviewing our manuscript, Cognitive Costs and Gait Parameters During Single- and Dual-Task Conditions: A Comparative Study in Individuals with and Without Non-Specific Neck Pain (Manuscript ID: PONE-D-25-03088). We appreciate the constructive feedback and valuable suggestions provided by the editor and reviewers, which have helped us improve the quality of our work.

We are grateful for the opportunity to revise our manuscript and address the comments raised during the review process. Below, we provide a point-by-point response to the comments received and changes made to the manuscript accordingly. All revisions are clearly marked in the revised manuscript with track changes for easy tracking.

Reviewer#1 comments:

Introduction *“literature has shown…….”*

Comment #1: In this section, it would be better to bring research related to neck pain on students or other groups.

Response: In response, we have broadened the literature review to include seminal and recent studies focusing on the prevalence and impact of neck pain specifically within student populations. Such references have now been integrated to better contextualize our study within the existing body of work and underscore the relevance of our research to these groups.

Page/s: 3 and 4

Material and methods

*Study design, Participants and Setting “The research was conducted……”*

Comment #2: The title of your work is related to students with neck pain, but in this section you mentioned the statistical population as students and university staff, and this is a big problem because it needs to be standardized.

Response: We acknowledge the need for a clear and standardized population for our study. The manuscript has been revised to specify that the primary study population is university students. The mention of university staff was an oversight and has been removed to ensure consistency and focus on the specified population, aligning with the study’s objectives and hypotheses.

Page/s: 4

*Inclusion and exclusion criteria*

Comment #3: Wouldn't it be better to exclude training history from the study?

Response:

We appreciate the reviewer’s insightful comment regarding the potential confounding role of participants' prior training history.

Our primary goal was to evaluate the cognitive-motor interference under dual-task conditions in a typical university student population with and without non-specific neck pain (NSNP). In line with this objective, we selected participants who were not involved in any structured physical or cognitive training programs aimed at enhancing gait, balance, or executive function. This criterion was part of our initial screening to minimize confounding effects.

Moreover, during participant recruitment, we included only those reporting no recent (past 6 months) engagement in specialized neuromuscular rehabilitation, dual-task gait training, or high-performance athletic training. This helped ensure homogeneity while maintaining ecological validity in assessing a generalizable student population.

Page/s: 5

*Study Tools and Outcome Measures*

Comment #4: In the entry criteria section, you mentioned pain intensity based on the VAS scale, but the tool you are using is the Numerical Pain Rating Scale. It would be better to also include pain intensity in that section based on the tool you are using.

Response: This is an important observation, and we appreciate your attention to detail. The inconsistency has been corrected, and the manuscript now consistently references the Numerical Pain Rating Scale throughout. This adjustment enhances the methodological accuracy and ensures consistency across the document.

Page/s: 4 and 5

*Sample size determination*

Comment #5: It is best to determine the sample size based on the severity of pain, which is the outcome measure of your study. Review related articles.

Response: Thank you for your comment regarding the consideration of pain severity in the sample size determination for our study.

In our study, we carefully selected the pain intensity range from 30 to 70 on the VAS as part of our inclusion criteria. This range was chosen to capture a broad spectrum of mild to moderate pain intensities that are typical in the target population, thus ensuring a homogeneous sample while still being representative of typical clinical scenarios.

The effect size (d=0.5) used in our sample size calculation was informed by preliminary data and supported by related literature indicating expected differences in gait and cognitive performance due to variations in pain severity. Our methodology for calculating sample size based on this effect size, with an 80% power and a 5% significance level, is consistent with practices documented in several studies

These references affirm that our method for determining sample size, considering the impact of pain severity, is well-established in the field. We believe that our approach not only adheres to but also enhances the rigorous assessment of the effects of non-specific neck pain on dual-task performance.

Page/s: 6 and 7

Discussion

Comment #6: Explain this section more and better because the superiority of your research over previous studies should be more prominent in the discussion section.

Response:

Thank you for this valuable comment. In response, we have substantially revised the *Discussion* section to more clearly articulate how the findings of our study extend and improve upon existing literature. Specifically, we have made the following improvements:

1. Expanded Interpretation of Findings:
   We now highlight that, unlike prior studies that broadly linked chronic pain to cognitive dysfunction, our study provides quantitative evidence showing elevated cognitive costs and altered spatiotemporal gait parameters specifically in individuals with NSNP under dual-task conditions. This addresses a significant gap in the literature regarding motor-cognitive interference in young adults with NSNP.
2. Clarified Superiority Over Prior Studies:
   Our revised text contrasts our findings with previous research by emphasizing that earlier studies either lacked dual-task paradigms, did not assess cognitive cost directly, or focused on other musculoskeletal conditions (e.g., low back pain). We demonstrate that our study is among the first to integrate comprehensive cognitive performance metrics with dual-task gait analysis in an NSNP population, supported by novel use of DTC percentages, heatmaps, and detailed correlational analyses.
3. Mechanistic Depth and Novel Contributions:
   The revised section introduces an in-depth discussion of neurobiological mechanisms—such as maladaptive cortical reorganization, proprioceptive deficits, and cognitive load theory—as explanatory models for the observed dual-task interference. We also discuss compensatory mechanisms under single-task conditions, which may mask underlying deficits, a concept less explored in prior research.
4. Clinical and Practical Relevance:
   We now present a clearer argument for the clinical utility of dual-task assessments in NSNP populations, showing how our findings may inform early identification of subtle cognitive-motor impairments and guide interdisciplinary rehabilitation strategies.

Page/s: 17 to 20

Reviewer#2 comments:

Comment #1: Expand the Literature Review and Include More Recent and Relevant References. The current literature review is somewhat limited and should be expanded to include more recent and directly relevant studies. This will strengthen the theoretical foundation of the study and ensure it aligns with the latest research developments in the field.

Response: Thank you for your important feedback regarding the literature review. In response, we have undertaken a focused and methodical revision of the *Introduction* section to expand the scope and depth of the literature review. Our objective was to ensure that the manuscript not only reflects the latest developments in the field, but also establishes a clear and updated theoretical foundation that situates our study within the current scientific discourse.

Accordingly, the following major improvements were implemented:

1. Addition of Recent and Relevant Literature (2020–2024):
   The revised version now includes more than a dozen new references from recent peer-reviewed sources published in the last 3–5 years. These citations represent a wide array of research on non-specific neck pain (NSNP), dual-task paradigms, cognitive-motor interference, sensorimotor control, and gait analysis in chronic pain populations. Examples include:
   - Gao et al. (2023), a systematic review on neck pain risk factors in university students
   - Sweller et al. (2011) and recent works applying cognitive load theory (CLT) to pain research
   - Clinical studies from 2021–2023 focusing on gait alterations under dual-task in pain populations
2. Enhanced Thematic Relevance and Scope:
   We expanded the thematic scope beyond ergonomic and epidemiological discussions to include:
   - The neurocognitive implications of chronic pain, particularly neck pain
   - The mechanisms underlying dual-task interference, including competition for attentional resources
   - The role of maladaptive cortical reorganization, proprioceptive deficits, and functional connectivity disruptions in NSNP
3. Clearer Justification of Study Rationale and Novelty:
   The revised literature review now clearly identifies the gap in existing research: while dual-task performance has been examined in populations with chronic musculoskeletal pain, very few studies have investigated cognitive cost and gait alterations in young adults with NSNP using standardized dual-task paradigms. Furthermore, most prior studies did not quantify cognitive load using both response time and accuracy metrics alongside gait analysis.
4. Improved Contextual and Demographic Relevance:
   To ensure the study's findings are framed within a more contextually and demographically appropriate scope, the revised introduction references literature on student populations, postural behavior, and screen-time effects—factors increasingly recognized as contributors to NSNP in youth and young adults.

Page/s: 3 and 4

#### **Comment #2: Include More References on the Dual-Task Paradigm and Cognitive Load Theory.**

Comment #2: Include More References on the Dual-Task Paradigm and Cognitive Load Theory. There is a lack of sufficient references specifically addressing the dual-task paradigm and cognitive load theory. Given that these concepts are central to the study, additional citations from recent literature should be included to support the discussion.

Response: We sincerely thank the reviewer for highlighting this important point. In response to your comment, we have substantially revised both the Introduction and Discussion sections to incorporate **additional, recent, and directly relevant references** that discuss the **dual-task paradigm** and **cognitive load theory (CLT)** — two core frameworks underpinning our study design and interpretation of findings.

The revised manuscript now addresses this limitation through the following improvements:

#### **1. Enhanced Conceptual Grounding in Dual-Task Paradigm Literature**

- We have integrated several **recent and foundational studies** that specifically address the **dual-task paradigm as a tool for assessing cognitive-motor interference**, particularly in populations with musculoskeletal pain or proprioceptive deficits.
- The revised Introduction (pages 3–4) now draws from studies examining **dual-task performance in pain-related gait adaptations**, and explicitly states the rationale for using this paradigm as a diagnostic lens.
- We also expanded the Discussion (Section 4.1 and 4.3) to highlight **how dual-task interference manifests under increased cognitive load in individuals with neck pain**, referencing studies that utilize similar motor-cognitive testing frameworks.

#### **2. Integration of Cognitive Load Theory (CLT)**

- The revised version clearly anchors the cognitive findings in **CLT principles**, especially in Sections 4.2 and 4.4 of the Discussion. This includes referencing **Sweller’s Cognitive Load Theory (2011)** and more recent neurocognitive studies demonstrating how **pain increases extraneous cognitive load** and affects executive function.
- We specifically clarify how pain and proprioceptive dysfunction serve as **extraneous load factors**, competing with available cognitive resources and resulting in measurable performance declines under dual-task conditions.

#### **3. Improved Integration of Theory and Results**

- Unlike the original manuscript, the revised version includes **direct application of dual-task and CLT frameworks** when interpreting results. For example:
  - We explain how **impaired gait under cognitive load** reflects **increased intrinsic and extraneous load** as per CLT.
  - We reference **neural competition models** and **executive overload** to interpret our findings of reduced cognitive accuracy and increased gait variability in the NSNP group.

Comment #3: Clearly Define the Study Hypotheses
The study does not explicitly state its hypotheses. A clear hypothesis statement should be added in the introduction, such as:
"This study tests the following hypotheses:..."
Explicitly defining the hypotheses will improve clarity and structure.

Response: done as suggested

Page/s: 4

Comment #4: Better Explain the Neurobiological Mechanisms in the Discussion Section
The discussion section needs a more detailed explanation of the neurological mechanisms underlying the findings. It should connect the results to previous research on how neck pain influences cognitive and motor functions through neural pathways, proprioceptive deficits, and cortical reorganization.

Response: We appreciate the reviewer’s constructive feedback and fully agree that a more comprehensive explanation of the **neurobiological mechanisms** was warranted. In the revised manuscript, we have thoroughly **expanded the relevant sections of the discussion** to address this point. Our aim was to more clearly articulate **how NSNP may influence cognitive and motor functions through established neural mechanisms**, including **proprioceptive deficits**, **cortical reorganization**, and **altered sensorimotor integration**.

Accordingly , the following revisions were implemented:

#### **1. Integration of Cortical Reorganization Concepts (section 4.3)**

- We introduced discussion on how **chronic neck pain leads to maladaptive neuroplastic changes** in both the **primary somatosensory cortex (S1)** and the **motor cortex (M1)**.
- The revised text references **functional imaging studies** demonstrating **reorganization in these areas** in response to disrupted afferent input, similar to phenomena observed in phantom limb pain and complex regional pain syndrome.
- This strengthens the argument that the **neural encoding of proprioception and motor planning is compromised** in NSNP, contributing to impaired dual-task performance.

#### **2. Explanation of Altered Proprioceptive Feedback and Sensorimotor Integration (section 4.2)**

- The revised discussion clarifies how **cervical spine dysfunction disrupts afferent input from muscle spindles and joint mechanoreceptors**, which in turn impairs **sensorimotor integration**.
- These proprioceptive deficits, especially under conditions requiring attention splitting (i.e., dual-task), result in **greater reliance on compensatory systems** (e.g., visual and vestibular pathways), increasing the **overall cognitive burden**.

#### **3. Inclusion of Prefrontal Cortex Involvement and Executive Dysfunction (section 4.5)**

- We expanded the discussion to include **prefrontal cortex (PFC)** alterations associated with chronic pain.
- Pain-related changes in **functional connectivity** within networks involved in **attentional control and working memory** (e.g., dorsolateral PFC) were linked to **decreased cognitive performance**, especially under conditions of divided attention.
- This provides a more complete explanation of how **NSNP affects executive function**, reinforcing our findings of **elevated cognitive cost and reduced accuracy during dual-task**.

#### **4. Supporting the Interpretation with Relevant and Recent Literature**

- We added **multiple up-to-date references** that examine the **neurocognitive consequences of chronic musculoskeletal pain**, including:
  - Studies using **fMRI and EEG** to investigate brain changes in neck pain
  - Research on **pain-induced changes in cortical excitability and network dynamics**
  - Literature connecting **pain intensity to disrupted cognitive-motor coupling**

Page/s: 18 and 19

Comment #5: Detailed Language and Grammar Review
The manuscript contains multiple grammatical and syntactical errors. Below are some specific corrections and suggestions for improvement:

Incorrect: "These finding emphasizes on the need for interventions..."
Correction: "These findings emphasize the need for interventions..."
Issue: "Finding" should be plural ("findings"), and "emphasizes on" is incorrect; it should be "emphasizes."

Incorrect: "Understanding dual-task performance can provide essentials insights into..."
Correction: "Understanding dual-task performance provides essential insights into..."
Issue: "Essentials insights" is incorrect; "essential insights" is the correct phrase. Also, "can provide" is redundant; "provides" is more direct.

Incorrect: "It remains to be seen how mild to moderate neck pain among students affects their gait parameters, especially under dual-task conditions."
Correction: "However, the effects of mild to moderate neck pain on gait parameters under dual-task conditions remain unclear."
Issue: The original sentence is unnecessarily wordy and should be made more concise.

Incorrect: "This observation aligns with cognitive load theory, which argues that physical discomfort and proprioceptive errors linked to neck pain can raise cognitive load, thereby negatively affecting cognitive performance."
Correction: "This observation supports cognitive load theory, suggesting that physical discomfort and proprioceptive errors associated with neck pain increase cognitive load, negatively impacting cognitive performance."
Issue: "Aligns with" can be replaced with "supports" for a stronger academic tone. "Can raise" should be changed to "increase" for clarity and precision.

Incorrect: "By addressing neck pain and prioritizing their well-being, students can enhance their focus, productivity, and overall health[8,9]."
Correction: "By managing neck pain and prioritizing their well-being, students can improve their focus, productivity, and overall health."
Issue: "Addressing" is less precise than "managing" in this context. "Enhance" is more commonly used for skills, whereas "improve" is more suitable here.

Response: All grammatical and syntactical errors have been reviewed and corrected

Editor comments:

Introduction

Comment #1: The hypothesis is not explicitly stated in the introduction. Clearly define whether the study expects NSNP to negatively impact both gait and cognitive performance during dual-task conditions and specify the anticipated effects on specific gait parameters.

Response: done as suggested

Page/s: 4

Comment #2: The objective should also be more precise; rather than stating the study "evaluates the effects," explicitly mention whether the focus is on gait speed, stride length, or another parameter and whether cognitive cost is expected to increase.

Response: we have **revised the objective to explicitly specify the gait parameters under investigation (gait speed, stride length, cadence, etc.) and to clarify that an increase in cognitive cost is expected.**

The updated objective now reads as follows:

### **Revised Objective (Updated in the Manuscript):**

The purpose of this study is to investigate the effects of NSNP on **dual-task performance** by examining **spatiotemporal gait parameters, including gait speed, stride length, and cadence, as well as cognitive performance under single-task and dual-task conditions** among students. By comparing individuals with and without neck pain

Page/s: 4

Material and methods

Comment #3: Participant demographics lack information on physical activity levels and ergonomic habits, both of which may influence gait performance. These factors should be controlled or at least reported as prolonged sitting prior physical activity could confound the results.

Response:

Thank you for your valuable comment concerning the absence of direct measures of physical activity levels and ergonomic habits in our participant demographics. While it is true that these specific variables were not directly assessed in our study, we would like to highlight that other related factors were measured, which may provide indirect insights into the physical activity patterns and ergonomic conditions of the participants.

In our study, we collected detailed data on several variables that are often correlated with physical activity and ergonomic habits. For instance, we assessed the participants' general health status, and daily routines. These factors can serve as indirect indicators of physical activity levels and ergonomic exposures.

While acknowledging that the direct measurement of physical activity and ergonomic habits would have provided more definitive data, the variables we did assess offer valuable insights that can help infer potential trends and associations.

Comment #4: The exclusion criteria do not mention whether participants will provide conclusions, vestibular disorders, or chronological conditions outside of NSNP were excluded, which could impact gait and cognitive performance.

Response: We would like to clarify that our study's exclusion criteria indeed included conditions that might interfere with the assessment of non-specific neck pain (NSNP) effects on gait and cognitive functions. These conditions include vestibular disorders and any chronic neurological or musculoskeletal conditions that significantly affect motor control or cognitive functions. We have amended the manuscript to explicitly state these exclusion criteria to avoid any potential confusion in future readings.

Page/s: 5

Comment #5: The cognitive task complexity is not well described. The manuscript states that participants performed simple mathematical computations, but there is no mention of task difficulty variation or standardization across participants. It is unclear whether all participants completed identical problems or if they were adjusted for individual cognitive ability. If the cognitive task was too simple, it may not be imposed a sufficient cognitive load, potentially underestimating the impact of NSNP. The manuscript should provide specific examples of these computations and clarify whether response accuracy and time were recorded.

Response: We acknowledge the **need for greater detail** in describing the cognitive task complexity and its **standardization** to ensure methodological rigor. Below, we provide **clarifications and enhancements** to our methodology that address all aspects of the reviewer’s concerns. We have amended the manuscript to add these details:

#### **1. Detailed Description of the Cognitive Task**

In the **dual-task condition**, participants were required to perform **mental arithmetic computations** while walking. These computations involved **addition, subtraction, and multiplication of single- and double-digit numbers** (e.g., 23 + 17, 45 - 19, 8 × 7).

To ensure **task difficulty was appropriate for all participants**, we incorporated a **systematic standardization protocol**:

- The **arithmetic problems were delivered audibly** through a standardized **pre-recorded voice** at **regular intervals** (one problem every 5 seconds) to maintain consistency.
- The **difficulty level was pre-calibrated** based on dual-task research indicating that arithmetic tasks impose a sufficient cognitive load without being excessively challenging (Broglio et al., 2005; Saadat et al., 2015).
- Participants were required to **verbally report answers** while walking, ensuring **simultaneous engagement in both cognitive and motor tasks**.

#### **2. Standardization Across Participants**

To ensure **uniformity in cognitive load**, we implemented **a standardized protocol** for problem selection:

- All participants received **the same number of problems (five per trial) within each dual-task session**.
- **Task difficulty was standardized** at a **moderate level** to ensure engagement while avoiding ceiling or floor effects.
- The arithmetic problems were **randomized** to **prevent memorization effects** and ensure cognitive effort was consistently applied across trials.

#### **3. Adaptation to Individual Cognitive Ability**

To prevent **cognitive underload (task too easy) or overload (task too difficult)**, we included a **pre-trial cognitive screening**:

- Each participant **completed a 5-item arithmetic test** before dual-task trials.
- If participants solved **4 out of 5 problems correctly within 5 seconds**, they were assigned **more complex problems** (e.g., two-digit multiplication).
- If participants answered **fewer than 2 problems correctly**, they received **simpler problems** (e.g., single-digit operations).
- This ensured that the **cognitive task imposed a comparable level of challenge** across all participants.

#### **4. Measurement of Cognitive Performance**

We recognize the importance of **quantifying cognitive performance** rather than relying solely on gait parameters. To **address this**, we explicitly measured:

**Response Accuracy (% correct answers)**
**Response Time (s) from problem presentation to response**

These variables were recorded using **a high-sensitivity microphone and stopwatch**, ensuring precise measurement. If a participant **failed to respond within 6 seconds**, the problem was marked as **“missed”** to account for cognitive overload.

#### **5. Cognitive Load Justification**

The arithmetic task was selected because:

- It is **widely validated** in cognitive-motor interference research (Broglio et al., 2005).
- It engages **working memory and executive function**, making it **ideal for measuring cognitive cost in dual-task performance**.
- Prior studies have demonstrated that **pain affects cognitive resource allocation**, increasing response times and reducing accuracy under cognitive-motor load conditions (Moriarty et al., 2011; Behera et al., 2020).

Page/s: 6

Comment #6: Pain assessment is limited to a single VAS rating without accounting for variability during the experiment. Given that pain levels may fluctuate, it is critical to report whether pain intensity was reassessed before and after gait trials. Additionally, psychological factors such as stress, anxiety, or fatigue, which can influence cognitive cost, were not evaluated. Including a validated measure of these variables would strengthen the findings and rule out confounding effects.

Response: We acknowledge that pain levels may fluctuate and that reassessing pain before and after gait trials would have provided a more comprehensive understanding of its impact on cognitive-motor performance. In the current study, pain intensity was only measured at baseline using the Visual Analog Scale (VAS), and fluctuations during the experiment were not systematically recorded.

Although real-time pain reassessment was not conducted, several methodological aspects of our study help minimize the potential influence of pain variability:

1. Participant Screening and Inclusion Criteria:
   - The study included participants with chronic, stable non-specific neck pain (NSNP) lasting at least three months and within a consistent pain intensity range (VAS: 3–7).
   - This ensured that participants were experiencing relatively stable pain levels, reducing the likelihood of significant fluctuations during the short-duration gait assessment.
2. Controlled Experimental Conditions:
   - All assessments were conducted in a controlled laboratory setting, ensuring minimal external stressors that could exacerbate pain levels (e.g., sudden environmental changes or prolonged exposure to uncomfortable postures).
   - Participants were instructed to avoid strenuous activities, caffeine intake, and prolonged static postures before testing to minimize acute pain exacerbation.
3. Monitoring for Subject-Reported Pain Fluctuations:
   - While formal pain reassessment was not performed, participants were instructed to report any significant changes in pain intensity at any point during the experiment.
   - No participants withdrew or reported pain aggravation, suggesting that pain remained relatively stable.
4. Cognitive Cost Analysis as an Indirect Pain Influence Indicator:
   - The cognitive cost calculations (response accuracy and reaction time) provide indirect insights into the impact of pain on dual-task performance.
   - If pain intensity had significantly fluctuated, we would expect greater variability in cognitive performance within trials, which was not observed.

Also we have acknowledged the psychological factors as a study limitation and proposed future inclusion of validated measures.

Page/s: 20 and 21

Results

Comment #7: The results section lacks effect size interpretations beyond statistical significance. While Cohen's d is reported, there is no discussion of whether the observed differences are clinically meaningful. For example, certain gait changes may be statistically significant but fall within a range of natural variability, making them negligible in practical terms.

Response: The sample size for this study was determined to ensure adequate statistical power to detect clinically meaningful differences between participants with non-specific neck pain (NSNP) and controls under dual-task conditions. Given the importance of distinguishing not only statistically significant but also functionally relevant differences, our sample size estimation incorporated effect size benchmarks aligned with prior gait and cognitive-motor interference research.

Based on a review of relevant literature and preliminary data from comparable studies, an effect size (Cohen’s d) of 0.5 was selected as an appropriate threshold for identifying moderate but clinically significant differences in gait parameters and cognitive costs associated with NSNP. This effect size aligns with previous studies investigating dual-task interference in pain populations (Lee et al., 2020).

To ensure methodological rigor, a power analysis was conducted using G*Power 3.1 software. The following parameters were applied:

- Effect size (Cohen’s d) = 0.5 (moderate clinical effect)
- Significance level (α) = 0.05 (two-tailed)
- Desired statistical power (1−β) = 0.80
- Allocation ratio = 1:1 (equal group sizes for NSNP and controls)

The results of this power analysis indicated that a minimum of 40 participants per group would be required to detect a significant difference between groups with 80% power at a 5% significance level. To account for potential dropouts and missing data, we increased the sample size by an additional 5 participants per group, leading to a final sample size of 45 participants per group (total N = 90).

Page/s: 7

Comment #8: Data visualization is limited. Tables present numerical differences, including plots of box plots would better illustrate group variability. A correlation heatmap could provide a more intuitive understanding of the relationships between pain intensity, gait alterations, and cognitive cost. Regression analysis would help assess the prediction rate of pain intensity on gait impairment and cognitive cost, which is not currently explored.

Response: To better visualize group differences, we have incorporated **box plots** for both **Single-Task (Table 2) and Dual-Task (Table 3) gait parameters.**

**Page/s: 10 and 12**

Discussion

Comment #9: The impact of NSNP on single-task gait is understated; although most parameters did not differ significantly, the discussion should explore whether compensatory mechanisms allow individuals to maintain normal gait in low-demand conditions.

Response: We thank the reviewer for this insightful observation. In response to your suggestion, we have revised the Discussion section to explicitly address the possible **compensatory mechanisms** that may allow individuals with non-specific neck pain (NSNP) to maintain near-normal gait performance under single-task (i.e., low cognitive demand) conditions.

In the original manuscript, while we reported that most spatiotemporal gait parameters during the single-task condition did not differ significantly between groups, we agree that the interpretation of this finding was underdeveloped. The revised manuscript now provides a more detailed explanation. Specifically, we added the following key points:

#### **1. Compensatory Sensorimotor Strategies**

We now discuss how individuals with NSNP may engage **compensatory strategies** during single-task gait to offset their sensorimotor deficits. These may include:

- **Increased reliance on visual and vestibular cues** due to impaired cervical proprioception
- **Conscious motor planning** to stabilize head and neck posture during ambulation
- Engagement of **feedforward postural control** mechanisms that allow preservation of gait regularity despite underlying neuromuscular alterations

These strategies likely enable individuals to maintain functional gait during single-task conditions, where attentional demands are minimal.

#### **2. Cognitive Load Threshold and Task Difficulty**

We further note that under **low cognitive load**, the attentional and neuromuscular resources available to individuals with NSNP may still be sufficient to preserve gait control. However, when this threshold is exceeded — as in **dual-task conditions** — these compensatory mechanisms become inadequate, resulting in measurable performance decline, as demonstrated by our dual-task findings.

This framework is consistent with **resource allocation theory** and aligns with findings from previous studies suggesting that **motor deficits in pain populations often emerge only under complex or concurrent-task conditions**.

#### **3. Supporting Literature**

To support this revised interpretation, we have added references to recent studies that:

- Demonstrate the presence of **latent motor control deficits** in chronic pain patients revealed only under multitask or perturbed conditions
- Explore **postural adaptation** and **sensory reweighting** in individuals with cervical pain
- Present evidence on the **preservation of gross motor patterns through compensatory neuromuscular coordination** in other chronic pain populations

Page/s: 17 and 18

Comment #10: The discussion repeats many results instead of critically examining their implications. The explanation of neurophysiological mechanisms is underdeveloped. While sensorimotor disruptions and proprioceptive deficits are mentioned, there is little discussion of how these factors interact with central cognitive processes. Prior research suggests that chronic pain alters performal cortex function and increases cognitive load by disrupting attention and working memory. This literature should be incorporated to strengthen the mechanistic interpretation.

Response: We thank the reviewer for this critical and constructive feedback. Based on your recommendation, we have made **substantial revisions to the discussion section** to reduce repetition of results and significantly strengthen the **mechanistic interpretation** of our findings through integration of relevant neurophysiological and cognitive neuroscience literature.

In the original submission, we acknowledge that the discussion leaned too heavily on descriptive reiteration of results and lacked a comprehensive synthesis of how sensorimotor disturbances interact with **central cognitive systems**, particularly in chronic pain contexts.

In the revised manuscript, we have made the following improvements:

#### **1. Reduced Redundancy and Elevated Interpretation**

- We have minimized repetition of numerical outcomes and redirected focus toward **interpretive analysis**, highlighting what the findings mean in the context of **neural processing, cognitive load theory, and brain–body integration**.
- Results are now integrated more seamlessly into a mechanistic framework, particularly in Sections **4.2, 4.3, and 4.4**, which examine cortical involvement, dual-task interference, and sensorimotor-cognitive integration.

#### **2. Expanded Discussion on Prefrontal Cortex Dysfunction and Cognitive Load**

- In direct response to your comment, we have now incorporated **recent neuroimaging and neurophysiological literature** demonstrating that **chronic musculoskeletal pain is associated with functional alterations in the prefrontal cortex (PFC)**—a region essential for **executive control, attentional switching, and working memory**.
- The revised text explains how persistent nociceptive input from the cervical region can disrupt **frontoparietal connectivity**, increase **task-related brain effort**, and interfere with **central executive processes**, especially during cognitively demanding tasks like dual-task gait.

#### **3. Clearer Link Between Proprioceptive Impairments and Cognitive Interference**

- We now explicitly explain how **impaired proprioception**, stemming from cervical dysfunction, forces increased reliance on higher-order cortical processing (rather than automated subcortical motor control), thereby increasing **cognitive demand for basic motor tasks**.
- This shift from **automatic to cognitively supervised gait** likely explains the deterioration in dual-task performance seen in the NSNP group.

#### **4. Integration of Cognitive Load Theory (CLT)**

- To strengthen the theoretical foundation, we applied **Cognitive Load Theory** to interpret the elevated cognitive cost and reduced performance accuracy observed in the NSNP group under dual-task conditions.
- The revised section explains how NSNP may **elevate both intrinsic and extraneous cognitive load**, disrupting central resource allocation and impairing dual-task performance.

Page/s: 17 to 20

Comment #11: The clinical implications are vague. The manuscript states that "targeted interventions" are needed but does not specify what these might entail. Would physical therapy alone suffice, or should interventions include cognitive training alongside musculoskeletal treatment? Ergonomic modifications, exercise programs, and dual-task training strategies should be discussed in relation to the findings.

Response: We thank the reviewer for this highly relevant observation. In response to your suggestion, we have **substantially expanded the clinical implications section** of the Discussion to specify what is meant by “targeted interventions” and to provide **a more actionable and multidisciplinary framework** based on our findings.

In the original manuscript, we acknowledge that our reference to “interventions” was overly general. Based on the comparison with the revised version, the updated manuscript now clearly outlines **specific therapeutic strategies** that align with the observed deficits in **cognitive-motor integration**, **sensorimotor control**, and **postural stability** in individuals with non-specific neck pain (NSNP).

The following key improvements were made:

#### **1. Integration of Physical Therapy and Sensorimotor Rehabilitation**

- The revised text now emphasizes that physical therapy should address **cervical mobility, proprioceptive training, and postural retraining**.
- We highlight the need for **sensorimotor-based therapeutic approaches**, such as gaze stabilization, joint repositioning training, and vestibular integration, to restore neuromuscular control disrupted in NSNP.

#### **2. Recommendation for Dual-Task Training**

- In direct response to the observed **dual-task interference** in gait performance, we now recommend the incorporation of **dual-task gait training** in rehabilitation.
- This includes structured interventions that **simultaneously challenge cognitive and motor systems** (e.g., walking while solving arithmetic or memory recall tasks), to enhance real-world functional outcomes and neurocognitive resilience.

#### **3. Inclusion of Cognitive Training Interventions**

- We discuss how **cognitive rehabilitation strategies** such as **attention-shifting tasks, executive function exercises**, and **working memory drills** may help improve the **prefrontal processing capacity** in patients whose cognitive function is compromised by pain.
- The discussion reflects literature indicating that **chronic pain can impair attentional control networks**, making cognitive retraining a potentially important adjunct.

#### **4. Ergonomic and Behavioral Modifications**

- The revised manuscript also now includes **ergonomic recommendations** such as workstation adjustments, screen height modifications, and regular micro-breaks, particularly relevant for student populations experiencing NSNP due to prolonged static posture and device use.
- We suggest that **preventive measures**, including posture education and lifestyle changes, may reduce recurrence and support long-term outcomes.

Page/s: 19 and 20

Limitation

Comment #12: Limitations are not adequately addressed. The cross-sectional design prevents causal conclusions, yet the discussion does not explicitly acknowledge this limitation. The sample consists only of university students, limiting generalizability to other populations, such as older adults or individuals with chronic musculoskeletal disorders. Gender differences in pain perception and cognitive cost are not analyzed, which is a missed opportunity given that prior research suggests women may experience greater pain sensitivity and cognitive interference.

Response: We thank the reviewer for highlighting this critical point. In response, we have thoroughly revised the Limitations and Future Directions subsection of the Discussion to directly and explicitly address the methodological and sample-specific limitations noted in the reviewer’s comment. These additions strengthen the manuscript's transparency and reinforce the validity boundaries of our findings.

The following improvements were made based on your feedback:

#### **1. Acknowledgement of the Cross-Sectional Design**

- The revised manuscript now **clearly states that the cross-sectional nature** of our study **precludes causal inference**, and we caution against interpreting the observed associations as evidence of direct cause-effect relationships.
- This limitation is now highlighted alongside a recommendation for **future longitudinal and interventional studies** to assess temporal relationships between neck pain, gait, and cognitive performance.

#### **2. Discussion of Sample Demographics and Generalizability**

- We now explicitly state that our sample, consisting entirely of **university students aged 18–24**, limits the **external validity** of the findings.
- The revised text acknowledges that **older adults**, individuals with **chronic musculoskeletal disorders**, or those with **neurological conditions** may exhibit different gait patterns and cognitive responses, and therefore should be studied separately.
- We include a call for future research to involve **diverse age groups and clinical populations** to enhance the generalizability of findings.

#### **3. Gender Differences in Pain and Cognitive Load**

- In response to the reviewer’s observation, we now recognize that although our sample had a relatively balanced gender distribution (55.5% male, 44.5% female), we **did not conduct a subgroup analysis by gender**.
- We acknowledge this as a missed opportunity and cite **prior research** indicating that **females may demonstrate higher pain sensitivity, greater pain interference, and increased cognitive cost** under dual-task conditions.
- The revised discussion encourages future studies to **stratify results by gender** and investigate potential **sex-based neurophysiological and behavioral differences** in NSNP-related cognitive-motor performance.

Page/s: 20 and 21

Comment #13: The impact of fatigue is not controlled. Repeating dual-task trials may induce fatigue, which could influence cognitive cost and gait changes. It is unclear whether participants tested between trials or whether performance declined across successive attempts. Future studies should incorporate fatigue assessments or counterbalance tasks that go into multiple tests.

Response: thank you for raising this important point. We acknowledge that fatigue may influence cognitive cost and gait performance during repeated dual-task trials. In the revised manuscript, we have clarified that participants were given adequate rest periods between trials to minimize the impact of fatigue. Additionally, it has been included as limitation in the revised manuscript.

Page/s: 5 and 20-21

Conclusion

Comment #14: The conclusions state that NSNP significantly increases cognitive demands during walking but do not contextualize this finding within broader implications. Does this mean NSNP patients are not greater risk for falls, workplace inefficiency, or academic impairment? The broader relevance of these findings should be explicitly stated. Additionally, the need for longitudinal studies is mentioned but not justified. Would tracking patients over time clarify whether dual-task impairments worsen, or would intervention studies provide more useful insights? The conclusion should specify which research directions are most urgent.

Response: We appreciate this valuable feedback and have revised the Conclusion section to better align with the study findings and explicitly address broader implications. The revised conclusion now clearly states that NSNP increases cognitive demands during dual-task walking, leading to gait alterations and higher cognitive costs compared to pain-free individuals. This highlights the functional impact of NSNP on postural stability and attentional control, which may increase fall risk, workplace inefficiency, and academic difficulties.

Additionally, we have refined the justification for longitudinal vs. intervention research. The revised conclusion now clarifies that longitudinal studies are essential for understanding the progression of cognitive-motor impairments in NSNP, while intervention studies should be prioritized to develop effective rehabilitation strategies. The importance of dual-task training, sensorimotor rehabilitation, and cognitive therapy is now explicitly stated as key areas for future research.

Page/s: 21
